# Effects of combined morbid insomnia and sleep apnea on long-term cardiovascular risk and all-cause mortality in elderly patients: a prospective cohort study

**DOI:** 10.1186/s12877-024-05147-2

**Published:** 2024-07-21

**Authors:** Fengfeng Fang, Zhihong Sun, Yinghui Gao, Jiming Han, Libo Zhao, Zhe Zhao, Zijun He, Zuo Zhang, Hongyan Bian, Lin Liu

**Affiliations:** 1 Department of Pulmonary and Critical Care Medicine of the Second Medical Center and National Clinical Research Center for Geriatric Diseases, Chinese PLA General Hospital, Beijing, China; 2 Department of Pulmonary and Critical Care Medicine of the Second Medical Center, Chinese PLA General Hospital, Beijing, China; 3 PKU-UPenn Sleep Center, Peking University International Hospital, Beijing, China; 4 Medical College, Yan’an University, Yan’an, China; 5 Cardiology Department of the Second Medical Center & National Clinical Research Center for Geriatric Diseases, Chinese PLA General Hospital, Beijing, China; 6 People’s Hospital of Ningxia Hui Autonomous Region, Beijing, China; 7 Cardiovascular and Cerebrovascular Disease Hospital of the Affiliated Hospital of Yan’an University, Yan’an, China

**Keywords:** Obstructive sleep apnea, Insomnia, COMISA, Adverse cardiovascular events, Mortality, Cardiovascular disease

## Abstract

**Purpose:**

It is reported that insomnia and obstructive sleep apnea (OSA) increase the incidence of adverse cardiovascular events. The aim of this study was to analyze the risk of cardiovascular disease and mortality in elderly patients with comorbid insomnia and obstructive sleep apnea (COMISA).

**Methods:**

We included 868 elderly patients with OSA who underwent sleep monitoring at a multicenter sleep room from January 2015 to October 2017. We collected demographic data, clinical features, medical history, sleep parameters, and laboratory findings. Cox proportional hazards analysis was used to identify the relationship between COMISA and adverse cardiovascular events and all-cause mortality.

**Results:**

There were 181 elderly patients with COMISA. The median follow-up was 43 months, during which we observed major adverse cardiac events (MACE) in 90 patients. The Kaplan-Meier survival curve indicated a significant relationship between COMISA and MACE (*P*_log Rank_ < 0.001). Multivariate Cox regression analysis showed that COMISA increased the incidence of MACE (HR = 2.328, 95% CI: 1.349–4.018, *P* = 0.002), hospitalization for unstable angina (HR = 2.915, 95% CI: 1.397–6.081, *P* = 0.004), and the combination of all events (HR = 2.301, 95% CI: 1.393–3.803, *P* = 0.001). However, there were no significant differences in cardiovascular death, all-cause mortality, myocardial infarction, or hospitalized heart failure in patients with COMISA (*P* > 0.05). Subgroup analyses showed that among COMISA patients, male sex (HR = 2.800, 95% CI: 1.458-5.377, *P* = 0.002), age < 70 years (HR = 4.050, 95% CI: 2.022–8.115, *P* < 0.001), and overweight and obesity (HR = 2.482, 95% CI: 1.383-4.453, *P* = 0.002) were associated with a higher risk of MACE.

**Conclusions:**

Our results showed that COMISA increased the risk of MACE, unstable angina, and the compound occurrence of all events. Male, overweight or obese COMISA patients under 70 years of age have an increased risk of MACE.

**Supplementary Information:**

The online version contains supplementary material available at 10.1186/s12877-024-05147-2.

## Introduction

Sleep disorders—insomnia and obstructive sleep apnea (OSA)—are often regarded as opposite clinical conditions, but they often occur together. OSA causes repeated closure or narrowing of the upper airway, resulting in snoring, sleep fragmentation, hypoxia, and poor sleep quality during sleep, which are considered factors in daytime sleepiness, with a prevalence of 10–20% in middle-aged and older adults [1]. Previously, insomnia was considered a secondary symptom of other diseases. However, in 2005, the National Institutes of Health considered insomnia to be an independent disorder coexisting with other diseases [2]. The symptoms of insomnia include difficulty starting or maintaining sleep, waking up early, and having difficulty resuming sleep and sleep impairments during the daytime [3]. It is closely related to nocturnal hyper-awakening. Due to the diagnostic criteria for insomnia have changed, European guidelines for the diagnosis and treatment of insomnia reported that the prevalence of insomnia ranges from 5.7 to 31.2% [4].

In 1973, Guilleminault proposed that insomnia and sleep apnea occur together as “comorbid insomnia and obstructive sleep apnea” (COMISA) [5], which is characterized by repeated apnea during sleep. The depth of breathing after apnea causes generalized awakening, often full awakening, resulting in sleep disorders that make it difficult to continue sleep. Thus, COMISA has both the sleep characteristics of OSA and the clinical features of insomnia [6]. This clinical syndrome has attracted much attention from researchers. According to Ong et al. [7], 6–84% of patients with OSA also suffered from insomnia, whereas OSA was associated with insomnia in 7–69% patients. Compared with only insomnia or OSA, COMISA patients have accumulated and substantial impairments manifesting as physical disorders (such as cardiovascular and cerebrovascular diseases), psychiatric disorders (such as mood and cognitive impairment), sleep, daytime function, and quality of life [8–11]. Of note, the risk of death and major adverse cardiac events (MACE) is higher in COMISA patients [12, 13]. According to the symptoms and complications of OSA, studies have described three groups of OSA symptoms, including mild symptoms; sleep disorder; excessive sleep. Among them, the proportion of sleep disorder group (32.7%) with insomnia and restless sleep as the main characteristics was higher than that of other groups [1, 14]. Therefore, COMISA was also considered to be a common OSA phenotype.

Indeed, its true burden is largely underestimated, and it is very challenging for medical staff to diagnose and manage it clinically. There have been some studies on COMISA in the past, but few studies on the occurrence of MACE. Therefore, this study investigated the proportion of subjects with OSA and insomnia to further explore their risk of MACE events. We hypothesized that OSA patients with insomnia were at higher risk of developing MACE.

## Methods

### Population

**Fig. 1:**
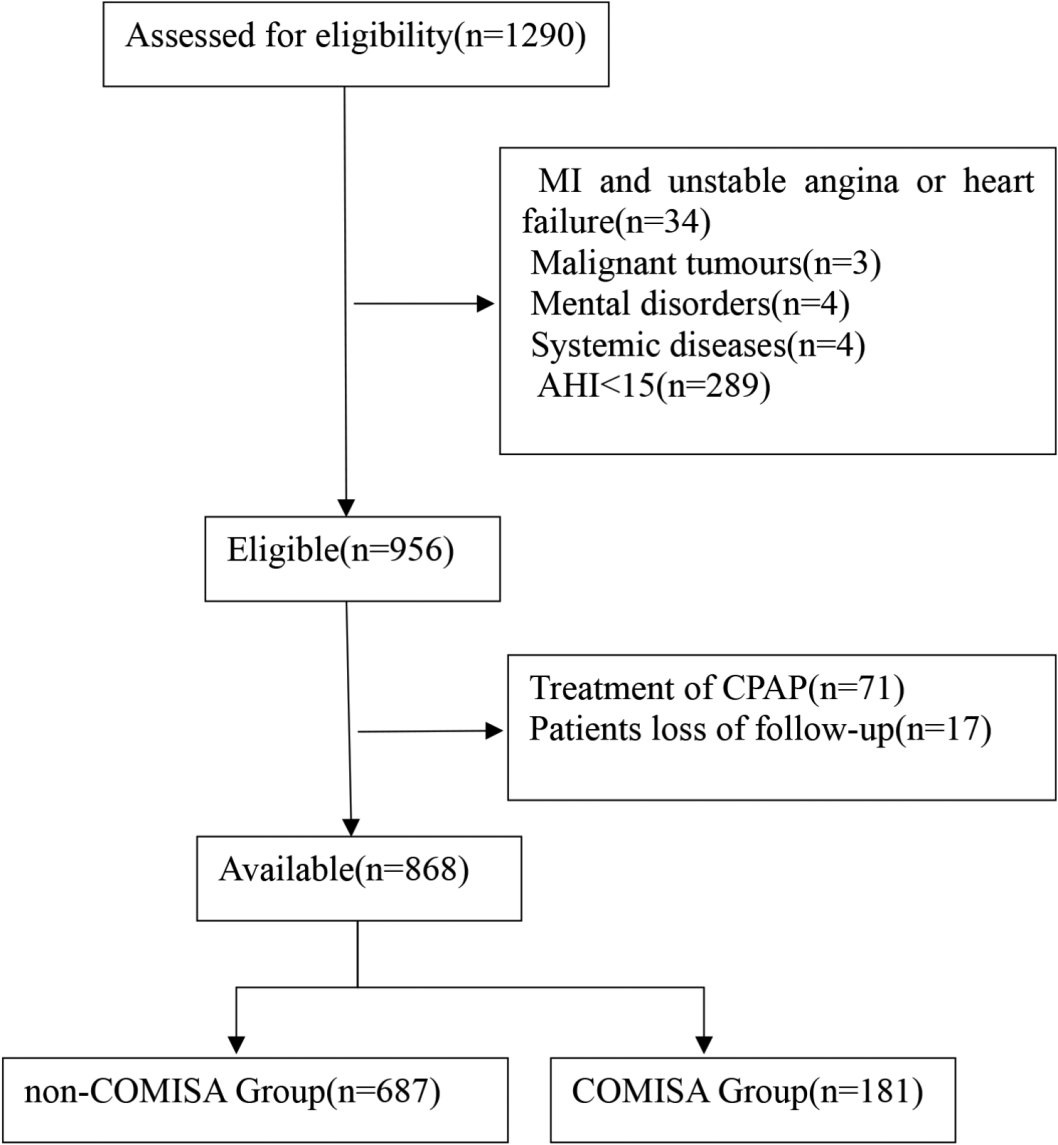
Study flowchart

This is a multicenter, prospective cohort study in which we recruited 1290 elderly patients diagnosed with OSA for the first time by polysomnography (PSG), from January 2015 to October 2017, at six sleep centers in Beijing and Gansu, China. All individuals who participated in this study were OSA patients aged ≥ 60 years, had accepted overnight PSG, had voluntarily participated in the study and signed an informed consent form, and were diagnosed as OSA patients with an apnea-hypopnea index (AHI ≥ 15 events/h. Exclusion criteria were those who had been i) diagnosed with myocardial infarction, ii) hospitalized due to unstable angina or heart failure, iii) had a history of malignant neoplasms, mental disorders, systemic diseases, or were taking antipsychotic drugs, iv) had previously been diagnosed with OSA or continuous positive airway pressure therapy, or v) who had missed visits. The study flowchart is shown in Fig. 1. Finally, 868 elderly patients with OSA who met the study criteria were included in the study.

The study was conducted in accordance with the principles of the Declaration of Helsinki and approved by the Ethics Committee of the PLA General Hospital (S2019-352-01).

### Polysomnography (PSG)

The participants were monitored overnight by a portable PSG machine (Compumedics, Melbourne, Australia) at the sleep center at 21:00 the same day. Standard PSG parameters including electroencephalography, electromyography, body position, nasal and oral airflow, respiratory effort, snoring, and pulse oxygen saturation were measured. Sleep stages, respiratory events (including apnea, hypopnea, and awakening) were manually calibrated, scored, and reviewed by two sleep physicians according to the American Academy of Sleep Medicine (AASM) guidelines [15].

The AHI was calculated as the number of apneas and hypopneas per hour of sleep. OSA was defined as AHI ≥ 5 events/hour. According to the AHI [16], the severity of OSA is classified as follows: mild: 5 events/h ≤ AHI < 15 events/h; moderate: 15 events/h ≤ AHI < 30 events/h; severe: ≥ 30 events/h.

### Covariates

On the second day of sleep monitoring, all recruits received laboratory tests. We obtained their clinical data from the hospital medical database: Demographic data: gender, age, height, weight, body mass index (BMI), systolic blood pressure (SBP), diastolic blood pressure (DBP), and self-reported history of smoking and drinking; Laboratory data include: fasting blood glucose (FPG), white blood cell count (WBC), absolute eosinophil value (EOS), etc.; Comorbidities: hypertension, coronary heart disease (CHD), cerebrovascular disease(CVD), carotid atherosclerosis , diabetes, atrial fibrillation (AF); and sleep parameters: total sleep time (TST), apnea low ventilation index (AHI), oxygen reduction index (ODI), the duration of time with SaO_2_ < 90% (TSA90), average oxygen saturation (MSpO_2_), minimum oxygen saturation (LSpO_2_) and other parameters. The data were collected by three experienced clinicians.

### Diagnostic criteria

The body mass index was calculated based on body weight (kg) / height (m^2^). Blood pressure was measured by standard requirements. The history of smoking was defined as ≥ one cigarette per day, and the history of drinking alcohol was defined as drinking ≥ one week/time for at least half a year. Hypertension was defined as measuring mean SBP ≥ 140 mmHg or mean DBP ≥ 90 mmHg at least two consecutive times, or using antihypertensive drugs. Diabetes mellitus was determined by (1) self-reporting or the use of insulin or hypoglycemic drugs; (2) Symptoms of diabetes mellitus (typical symptoms, including polydipsia, polyuria and unexplained weight loss) or fasting plasma glucose ≥ 7.0 mmol/L (126 g/L). CHD, carotid atherosclerosis, AF and cerebrovascular disease were determined by relevant clinical diagnostic records.

Insomnia: Diagnoses based on ICSD-3 criteria [17] include at least one: incubation period > 30 min for falling asleep, waking up after falling asleep > 45 min, or sleep efficiency < 75%, and self-reported daytime injuries associated with insomnia symptoms.

Combined morbid insomnia and sleep apnea (COMISA) [18] : It was defined as the presence of OSA (AHI ≥ 15 events/h) and insomnia disorders that occurred simultaneously.

### Outcomes and follow-up

This study was a multicenter cohort study. The patient was diagnosed with OSA by PSG, followed by telephone and outpatient follow-ups by two specially trained investigators. Every six months, the patient was followed up to document clinical features, signs, and the occurrence of other conditions. Follow-up was up to December 2020, with a median follow-up of 43 months. The baseline data and entry of follow-up outcomes for follow-up participants were quality controlled and proofread by third parties. All patients were given standard care services during follow-up based on different underlying medical conditions. MACE, which included myocardial infarction, death from cardiovascular causes, and hospitalization for unstable angina or heart failure, was the primary endpoint. We also assessed all-cause death, the composite of all events, and components of the MACE as secondary endpoints. The study was considered terminated if a patient experienced a new MACE or all-cause mortality during follow-up—the patient’s first MACE or all-cause death. Two or more MACE events were counted as one event, reporting the first event time and event as the outcome. Patients’ survival status was determined based on outpatient records or information provided by their relatives. The patient’s medical history and corresponding diagnostic report who self-report MACE events must be verified by two physicians through the electronic medical record system of each hospital until the end of the study. The patient’s survival status was determined based on outpatient records or information provided by their relatives.

### Statistics

Continuous variables with a normal distribution are expressed in (mean ± standard deviation) or median (interquartile range); Categorical variables are expressed as relative numbers. According to whether or not insomnia was present, patients were divided into COMISA and non-COMISA groups. Differences in characteristics of two groups of samples were assessed using t-tests, Mann-Whitney U tests or Chi-square test. We used a Kaplan-Meier survival curve and log-rank testing to analyze the association between insomnia and the follow-up events. All events of COMISA were analyzed using the Cox proportional risk regression model. Model 1 was unadjusted; Model 2 further adjusted gender, age, BMI, hypertension, CHD, CVD, diabetes, AF, as well as AHI, ODI, TSA90, LSpO_2_, FPG, WBC, EOS. All data statistics and analysis were analyzed using SPSS 25.0 software, with *P* < 0.05 as statistically significant difference.

## Results

### Baseline characteristics

The study subjects consisted of 868 patients with OSA (Fig. 1). Among them, non-COMISA had 687 participants with an average age of 66.75 ± 6.61; COMISA had 181 participants with an average age of 68.50 ± 6.83. Compared with the non-COMISA group, the COMISA group had higher age, BMI, SBP; Sleep indicators AHI, TSA90 and ODI was higher, LSpO_2_ was lower; Hypertension, CHD, CVD, diabetic disease and AF history was higher; FBG, WBC and EOS was higher, the difference was statistically significant (*P* < 0.05) (Table 1).

**Table 1:** General characteristics of the study subject

	Non-COMISA (*n* = 687)	COMISA (*n* = 181)	*P*
Male	447(65.1)	99(54.7)	0.010
Age	65.00(61.00,70.00)	67.00(63.00,73.00)	<0.001
Height	168.00(160.00,172.00)	165.00(160.00,170.00)	0.061
Weight	74.25 ± 12.56	75.63 ± 13.92	0.133
BMI	26.70 ± 3.85	27.58 ± 4.20	0.013
SBP	130.00(123.00,140.00)	139.50(126.00,152.50)	<0.001
DBP	76.00(70.00,82.00)	75.00(70.00,83.00)	0.523
Smoking	164(23.9)	35(19.3)	0.197
Drinking	66(9.6)	24(13.3)	0.152
AHI (events/h)	31.10(22.00,48.40)	46.85(35.50,59.95)	<0.001
TST(h)	7.01(6.07,7.50)	7.00(6.03,7.48)	0.825
ODI (events/h)	26.00(15.15,43.90)	41.10(23.70,52.55)	<0.001
TSA90(min)	16.60(3.80,66.43)	24.36(6.08,107.60)	0.011
MSpO_2_(%)	93.00(92.00,95.00)	93.00(91.00,95.00)	0.116
LSpO_2_(%)	79.00(71.00,84.00)	76.00(65.00,82.00)	<0.001
Hypertension (n, %)	425(61.9)	138(76.2)	<0.001
CHD (n, %)	142(20.7)	72(39.8)	<0.001
CVD (n, %)	92(13.4)	61(33.7)	<0.001
Carotid atherosclerosis (n, %)	154(22.4)	49(27.1)	0.188
Diabetes (n, %)	149(21.7)	80(44.2)	<0.001
AF (n, %)	51(7.4)	28(15.5)	0.001
FPG (mmol/L)	5.69(5.04,6.40)	5.96(5.31,7.47)	<0.001
WBC (10^9/L)	6.21((5.27,7.22)	6.54(5.56,7.37)	0.039
EOS (10^9/L)	0.02(0.01,0.05)	0.03(0.02,0.05)	0.037

BMI, body mass index; SBP, systolic blood pressure; DBP, diastolic blood pressure; AHI, the apnea-hypopnea index; TST, total sleep time; ODI, the oxygen desaturation index; TSA90, the duration of time with SaO_2_ < 90%; MSpO_2_, the mean pulse oxygen saturation; LSpO_2_, the lowest pulse oxygen saturation; CHD, coronary heart disease; CVD,cerebrovascular disease;AF, atrial fibrillation; FPG, fasting plasma glucose; WBC, white blood cell; EOS, Eosinophil absolute values.

### Primary outcomes: MACE

**Fig. 2:**
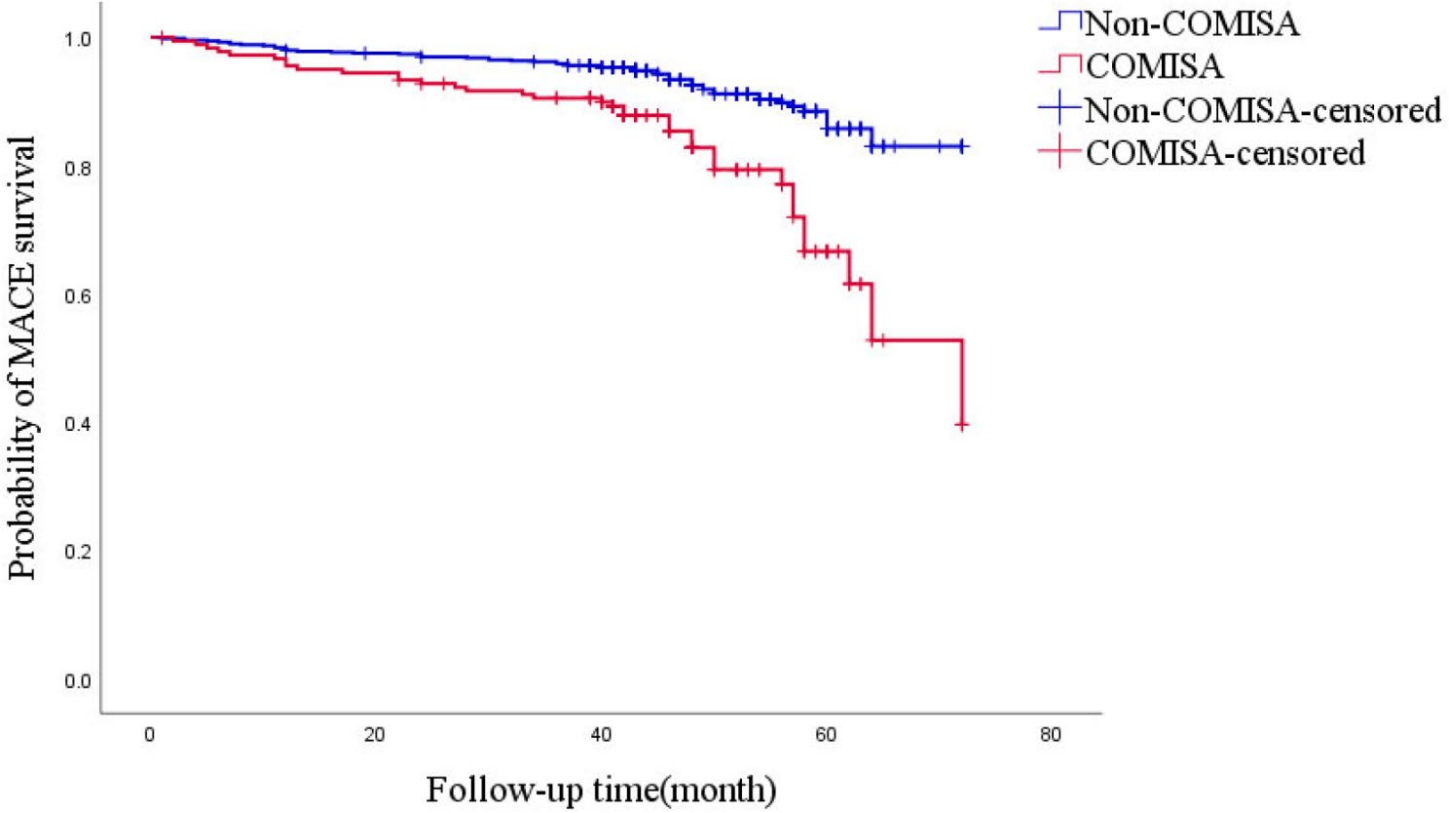
Kaplan-Meier estimates of cumulative incidence (%) for MACE. *P*_Log Rank_ < 0.001

In this study, 90 MACE events occurred over a median of 43 months (ranging from 6 to 72 months): 35 in patients with COMISA and 55 in patients without it. A Kaplan-Meier analysis revealed that COMISA patients with MACE had significantly more cumulative events than non-COMISA patients (*P*_Log Rank_ < 0.001), Fig. 2. Adjusted Cox proportional risk regression model, model 1 was unadjusted; Model 2 adjusts for gender, age, BMI, hypertension, CHD, CVD, diabetes, AF, and AHI, ODI, TSA90, LSpO_2_, FPG, WBC, EOS based on Model 1. Comorbidities of hypertension and CHD significantly increased the risk of MACE in patients with COMISA (HR = 2.328, 95% CI: 1.349–4.018, *P* = 0.002), Table 2. In subgroup analyses, COMISA patients who were male (HR = 2.800, 95% CI: 1.458-5.3771, *P* = 0.002), aged < 70 years (HR = 4.050, 95% CI: 2.022–8.115, *P* < 0.001), and overweight or obese (HR = 2.482, 95% CI: 1.383-4.453, *P* = 0.002) had a higher risk of MACE, Table 3.

**Table 2:** Relationship between COMISA and the incidence of all events

	Unadjusted analysis		Adjusted analysis	
	HR (95% CI)	*P*-Value	HR (95% CI)	*P*-Value
MACE	2.736(1.790–4.183)	<0.001	2.328(1.349–4.018)	0.002
Cardiovascular death	1.920(0.666–5.535)	0.227	1.113(0.254–4.868)	0.887
Myocardial infarction	2.956(1.458–5.992)	0.003	2.002(0.802-5.000)	0.137
Hospitalization for unstable angina	3.093(1.710–5.595)	<0.001	2.915(1.397–6.081)	0.004
Hospitalization for heart failure	1.795(0.463–6.962)	0.397	0.686(0.103–4.559)	0.696
All-cause mortality	2.279(1.188-4.370)	0.013	1.173(0.496–2.776)	0.716
Composite of all events	2.651(1.812–3.879)	<0.001	2.301(1.393–3.803)	0.001

Model 1 was unadjusted

Model 2 further adjusted gender, age, BMI, hypertension, CHD, cerebrovascular disease, diabetes, AF, as well as AHI, ODI, TSA90, LSpO_2_, FPG, WBC, EOS

**Table 3:** Subgroup analysis of the association between COMISA and MACE

	Unadjusted analysis		Adjusted analysis	
	HR (95% CI)	*P*-Value	HR (95% CI)	*P*-Value
Age				
< 70	4.100(2.358–7.129)	<0.001	4.050(2.022–8.115)	<0.001
≥ 70	1.478(0.755–2.890)	0.254	1.644(0.640–4.223)	0.301
Gender				
Male	3.340(1.995–5.591)	<0.001	2.800(1.458–5.377)	0.002
Female	1.930(0.910–4.091)	0.086	1.486(0.553–3.998)	0.432
BMI				
Normal (18.5–23.9)	2.113(0.568–7.866)	0.265	2.278(0.240-21.589)	0.473
Overweight and obese (≥ 24)	2.937(1.861–4.634)	<0.001	2.482(1.383–4.453)	0.002

Model 1 was unadjusted

Model 2 further adjusted gender, age, BMI, hypertension, CHD, cerebrovascular disease, diabetes, AF, as well as AHI, ODI, TSA90, LSpO_2_, FPG, WBC, EOS

### Secondary outcomes: all-cause Mortality, Components of MACE, and composite of all events

**Fig. 3:**
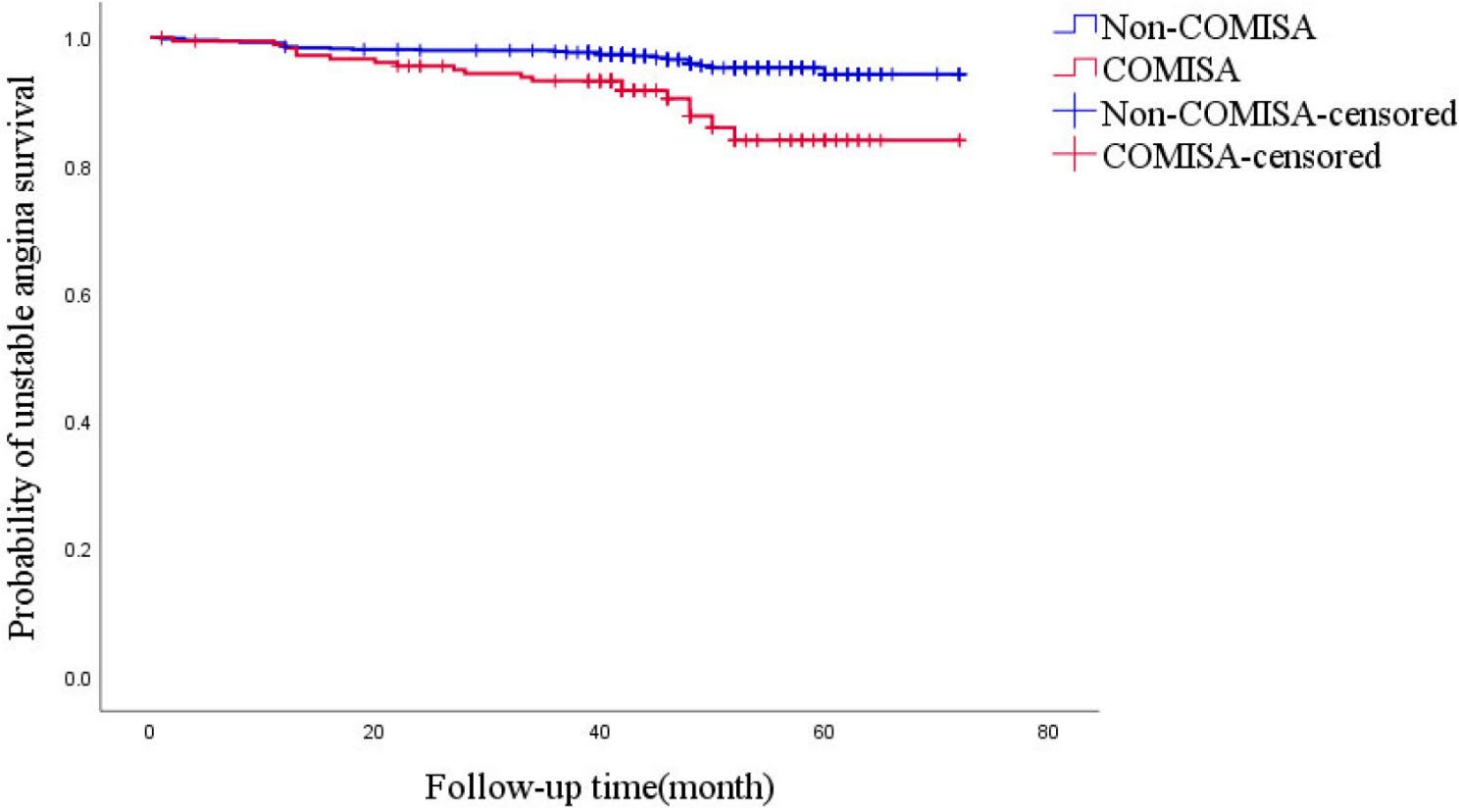
Kaplan-Meier estimates of probability of survival (%) for unstable angina. *P*_Log Rank_ <0.001

**Fig. 4:**
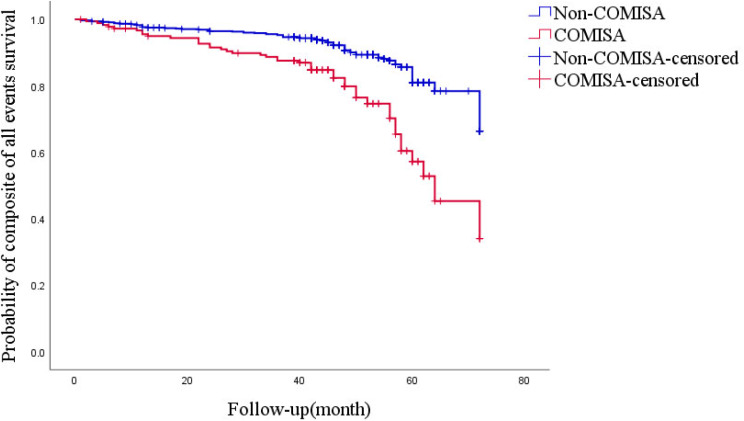
Kaplan-Meier estimates of probability of survival (%) for composite of all events. *P*_Log Rank_ <0.001

By the end of the follow-up period, 41 patients died, and 7.7% of those in the COMISA group died compared with 3.9% of those in the non-COMISA group. Cox univariate analysis showed higher risk of all-cause death in patients with COMISA (HR = 2.279, 95% CI: 1.188-4.370, *P* = 0.013). However, the risk of adjusted all-cause mortality was not significant (HR = 1.173, 95% CI: 0.496–2.776, *P* = 0.716), Table 2. Based on the Cox regression analysis-adjusted model, there was no significant difference in cardiovascular death, heart failure hospitalization, and all-cause death between COMISA and non-COMISA patients (*P* > 0.05). However, insomnia significantly increased the risk of unstable angina (HR = 2.915, 95% CI: 1.397–6.081, *P* = 0.004) and all events (HR = 2.301, 95% CI: 1.393–3.803, *P* = 0.001), Table 2. The Kaplan-Meier curve presented the relationship between insomnia and unstable angina and all events (*P*_Log Rank_ <0.001, *P*_Log Rank_ <0.001, respectively) (Figs. 3 and 4).

## Discussion

This is a prospective cohort study. Ultimately, we included 868 elderly patients with OSA, of whom 181 had COMISA at a prevalence of 20.9%. Notably, however, this differed from the findings of Lang et al. [8], who studied men aged 40–85 years with a COMISA prevalence of 6.7%. In epidemiological surveys [19], COMISA had a global prevalence of between 18% and 42%. The possible inconsistencies or broad scope of its prevalence are due to differences caused by the types of populations included in the studies and the differences in the diagnostic criteria for OSA and insomnia used in COMISA. OSA is a known risk factor for CVD, and insomnia also increases the risk of CVD [20]. Our results indicated that people with insomnia had a significantly elevated risk of MACE compared to people without insomnia. This was consistent with the results of Lechat [21], thus providing more evidence that COMISA patients had a higher risk of cardiovascular events. Additionally, the risk of all-event components increased by 2.3 times, and the risk of unstable angina requiring hospitalization increased by 2.9 times. We also looked at several subgroups of COMISA patients; there was a significantly greater association between the risk of MACE and overweight and obese men aged < 70 years.

At present, the pathologic mechanism of COMISA is not yet clear. It has been suggested that COMISA is caused by a bidirectional action between insomnia and OSA, which has been supported by several studies [22–24].First, OSA may be a risk factor for insomnia disorders. Repeated apneas or hypopnea during sleep cause sleep disruption and may be seen as awakening or sustained awakening [25], promoting maladaptive cognition of sleep, poor sleep quality, and increasing anxiety and alertness, thus leading to the development of insomnia. Second, insomnia can exacerbate or promote the development of OSA, which may increase susceptibility to episodes of apnea. Insomnia leads to a lower respiratory arousal threshold, activation of oxidative stress, increased sympathetic tone and inflammation, and disorders of endothelial function [23]. The over-awakened state of insomnia increases the propensity for short sleep, which in turn exacerbates the apnea [6]. In fact, COMISA is more common in patients with low wake-up thresholds or a tendency to awaken easily from respiratory stimuli than OSA alone [26]. Patients with low wake-up thresholds have hyperventilation during arousal, resulting in greater CO_2_ reduction and consequent worsening of upper airway muscle tone and OSA severity [24]. Finally, the two-way relationship between OSA and insomnia leads to more sleep fragmentation and sleep deprivation in patients, leading to worsening of the disease. Studies have confirmed [23] that the hypothalamic-pituitary-adrenal axis (HPA) pathway and metabolic factors are the physiological mechanisms that primarily act between them.

There is no doubt that OSA plays an important role in the development of numerous CVD and other conditions [27]. Similarly, insomnia increases the risk of CVD, such as hypertension [28], CHD [29], AF [30], heart failure [31], and cardiovascular death [20]. In our study, we discovered that COMISA patients had higher AHI and ODI, and lower LSpO_2_ than non-COMISA patients. Intermittent hypoxia reoxygenation, activation of oxidative stress and increased systemic inflammation, causing autonomic dysfunction and endothelial dysfunction, increased damage to the atria and large vessels, resulting in cardiovascular damage [20, 32, 33]. Furthermore, dysregulation of the HPA axis and glucose metabolism [34] associated with insomnia and neurocognitive-physiological wakefulness [35] leads to increased heart rate and blood pressure, dyslipidemia, impaired glucose metabolism [36], and promotes the occurrence of MACE events. Further, we found that the COMISA group had a higher prevalence of CVD, with a 2.3-fold higher risk of developing MACE than in the non-COMISA group. Among these MACE events, the proportion of unstable angina requiring hospitalization was relatively large. COMISA was strongly associated with the risk of developing unstable angina requiring hospitalization. There is evidence that nocturnal intermittent hypoxia is associated with a higher prevalence of CVD in COMISA [37]. According to a systematic review of a prospective cohort study [38], 122,501 participants without baseline CVD, but with insomnia, had a 45% higher risk of CVD or death over a follow-up period of 3–20 years. Another study [39]reported that among the 4437 participants without CVD at baseline, 818 were later observed to be diagnosed with CVD. There was a 29% higher risk of CVD in the insomnia group compared with the control group. It has been found [40, 41] that COMISA contributes more to CVD than OSA alone or insomnia alone. Therefore, our study has important guiding significance for the clinical occurrence of MACE events in COMISA patients.

Clinically, OSA is associated with all-cause mortality. OSA patients with moderate to severe AHI are more likely to have adverse outcomes, such as all-cause mortality [42]. In a population-based cohort study [43], 15,511 cohort respondents were followed for 14 years and insomnia was found to be associated with a greater risk of mortality from all causes. However, Bertisch et al. [39] reported that after adjusting for propensity, the insomnia or poor sleep quality with short sleep duration was associated with an increased risk of CVD, but not all-cause mortality. This was consistent with our results. In the initial unadjusted model, insomnia was significantly associated with all-cause death. Adjusting for confounding factors, however, did not show any significant impact. This may be because sleep disturbances and mortality depend on study design, including cohort age, insomnia diagnosis, differences in PSG and male-to-female ratios. While all-cause mortality can impact both clinical diagnosis and treatment of patients with insomnia and OSA, it cannot be ignored.

To our knowledge, this was the first study to report all-cause death, MACE, and the risk of all components among patients with COMISA. Studies have reported [44] sex-related differences in COMISA; OSA is more common in men, while women are more likely to have insomnia. Our findings suggested that male COMISA patients had a higher risk of developing MACE, which can be explained by hormone secretion. Women are protected from CVD damage by estrogen, which upregulates NO and activates eNOS rapidly to protect them from damage [45].Furthermore, men and women have different sleep stress responses, social behaviors, clock genes, and breathing patterns, all of which affect CVD susceptibility [19].

Our findings revealed that COMISA patients who were < 70 years old had a higher risk of MACE. This may be due to the peak prevalence of OSA in < 70-year-old people. In addition, sleep-wake symptoms have been reported to diminish with age [46, 47]. Older adults typically adjust their activity patterns, and insomnia may be better tolerated in older adults [48]. Alternatively, it could be the “paradox of happiness” in which older adults do not report dissatisfaction truthfully because their actual health exceeds expected levels, and/or they reduce their daily needs and tolerate less restorative sleep. This may lead to underreporting of insomnia. Finally, most sleep-wake symptoms occur in the seventh decade of life [49].

Patients with COMISA had higher BMI than patients with other symptoms [6]. Our data showed that the incidence of MACE was higher in overweight and obese COMISA patients. It is well known that obesity is a risk factor for OSA and insomnia. Overweight can lead to hemodynamic changes. Overweight and obesity activate the renin-angiotensin-aldosterone system [50], while sympathetic activity is also increased. Finally, CVD can be caused by obesity-related disorders such as inflammation, insulin resistance, endothelial dysfunction, and metabolic disorders [51, 52].

Our study had some limitations. First, the median follow-up was 42 months, which was short, and the endpoint event may not have completely occurred. Second, our study only assessed cardiovascular events and all-cause mortality in COMISA and non-COMISA groups, not include healthy controls. Also, we did not study sleep subtypes as part of the COMISA study, and there may be differences in the risk of MACE events between different insomnia subtypes. Despite these limitations, our study remained valuable. Finally, unmeasured factors, such as socioeconomic status, education or marital status, also play a role [1]. Individuals with low economic level had a higher risk of OSA, and treatment compliance was lower [53]. Stable economic status can improve treatment enthusiasm. People who were married or in a relationship were also more motivated to treat their illness than those who were single.

## Conclusion

We found that COMISA increases the risk of MACE in older patients who have unstable angina, and all of these events at the same time. According to subgroup analyses, men under 70 years of age who were overweight and obese had a greater risk of MACE. Comorbid insomnia or insomnia-like symptoms can increase the risk of MACE in patients with OSA, and identifying these patients is important for clinical personalized treatment.

### Electronic supplementary material

Below is the link to the electronic supplementary material.

Supplementary Material 1

## Data Availability

Our research is a teamwork. If everyone agrees to share the data, the first author or corresponding author can be contacted to obtain the information.
