# Supplementary material for: Effects of combined morbid insomnia and sleep apnea on long-term cardiovascular risk and all-cause mortality in elderly patients: a prospective cohort study
Source: BMC Geriatr. 2024 Jul 21;24:622. doi: 10.1186/s12877-024-05147-2 (PMC11265059; doi:10.1186/s12877-024-05147-2)
Supplement: Supplementary file 1 — Supplementary Material 1 [file 12877_2024_5147_MOESM1_ESM.docx]

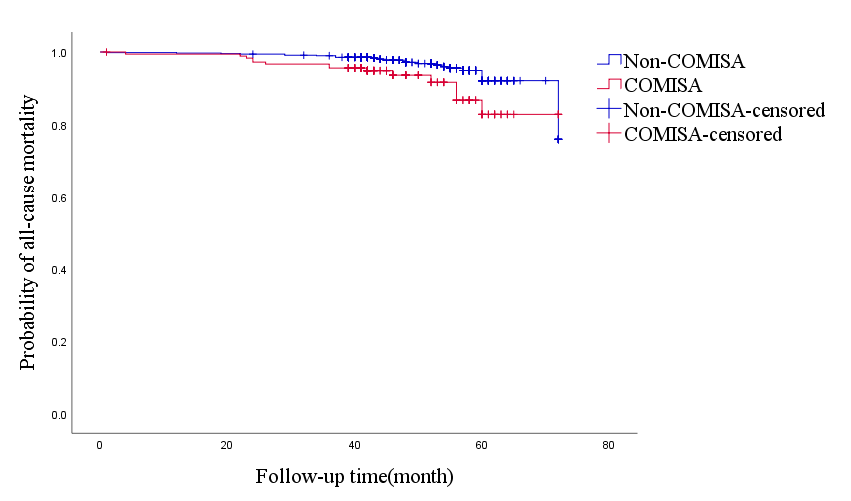


Supplementary Fig. 1 Kaplan-Meier estimates of probability of survival (%) for all-cause mortality. *P*_Log Rank_ = 0.007


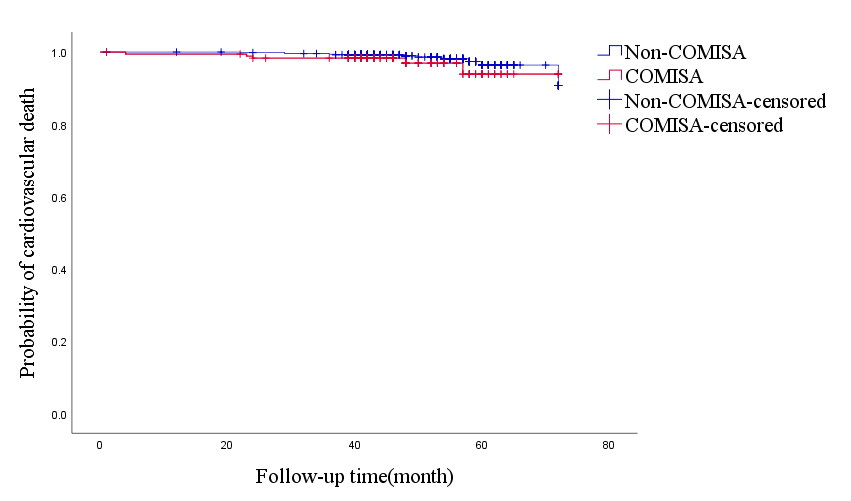


Supplementary Fig. 2 Kaplan-Meier estimates of probability of survival (%) for cardiovascular death. *P*_Log Rank_ = 0.227


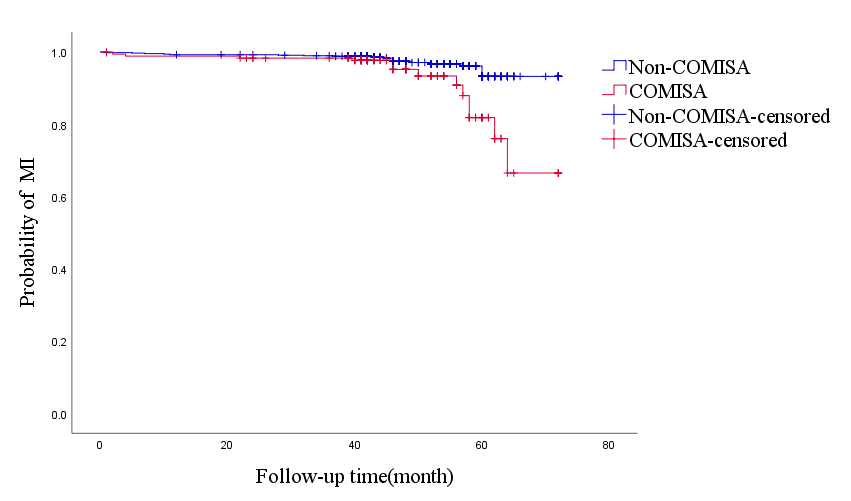


Supplementary Fig. 3 Kaplan-Meier estimates of probability of survival (%) for MI. *P*_Log Rank_ = 0.003


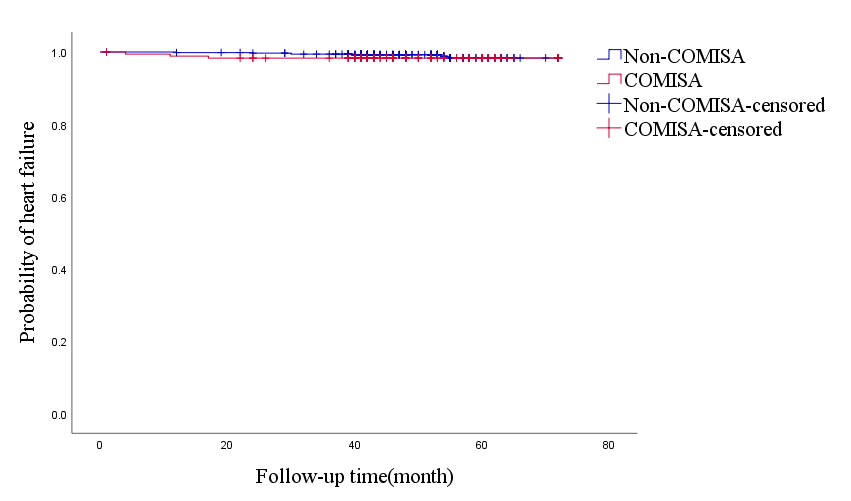


Supplementary Fig. 4 Kaplan-Meier estimates of probability of survival (%) for heart failure. *P*_Log Rank_ = 397
